# Olive Oil and Vitamin D Synergistically Prevent Bone Loss in Mice

**DOI:** 10.1371/journal.pone.0115817

**Published:** 2014-12-31

**Authors:** Camille Tagliaferri, Marie-Jeanne Davicco, Patrice Lebecque, Stéphane Georgé, Marie-Jo Amiot, Sylvie Mercier, Amélie Dhaussy, Alain Huertas, Stéphane Walrand, Yohann Wittrant, Véronique Coxam

**Affiliations:** 1 Institut National de la Recherche Agronomique (INRA), Unité Mixte de Recherche (UMR) 1019, Unité de Nutrition Humaine, Centre de Recherche en Nutrition Humaine (CRNH) Auvergne, Clermont-Ferrand, France; 2 Clermont Université, Université d’Auvergne, Clermont-Ferrand, France; 3 Lesieur, 29 quai Aulagnier, 92665 Asnières-sur-Seine cedex, France; 4 Biochemistry Department, Centre Technique de Conservation des Produits Agricoles (CTCPA), Site Agroparc, 84911 Avignon Cedex 9, France; 5 INRA, UMR 1260, Marseille, France; 6 Institut National de la Santé et de la Recherche Médicale (INSERM), UMR 1260, “Nutrition, Obésité et Risque Thrombotique”, Marseille, France; 7 Université d’Aix-Marseille, Faculté de Médecine, Marseille, France; Université de Lyon - Université Jean Monnet, France

## Abstract

As the Mediterranean diet (and particularly olive oil) has been associated with bone health, we investigated the impact of extra virgin oil as a source of polyphenols on bone metabolism. In that purpose sham-operated (SH) or ovariectomized (OVX) mice were subjected to refined or virgin olive oil. Two supplementary OVX groups were given either refined or virgin olive oil fortified with vitamin D3, to assess the possible synergistic effects with another liposoluble nutrient. After 30 days of exposure, bone mineral density and gene expression were evaluated. Consistent with previous data, ovariectomy was associated with increased bone turnover and led to impaired bone mass and micro-architecture. The expression of oxidative stress markers were enhanced as well. Virgin olive oil fortified with vitamin D3 prevented such changes in terms of both bone remodeling and bone mineral density. The expression of inflammation and oxidative stress mRNA was also lower in this group. Overall, our data suggest a protective impact of virgin olive oil as a source of polyphenols in addition to vitamin D3 on bone metabolism through improvement of oxidative stress and inflammation.

## Introduction

Osteoporosis, defined as “a systemic skeletal disease characterized by low bone mass and micro-architectural deterioration of bone tissue with a consequent increase in bone fragility and susceptibility to fracture” [1], is a leading cause of morbidity and mortality in the elderly. This is why, with an ageing population throughout much of the world, improving prevention of such a disease will play a major role in the sustainability and implementation of good personal and public health care. In this light, there is a growing interest among the medical scientists in the search of new strategies, including dietary approaches. Although calcium and vitamin D have been the primary focus of nutritional prevention, analysis of ongoing challenges has highlighted that the human diet contains, in addition to essential macronutrients, a complex array of naturally occurring bioactive micronutrients that may confer significant long term health benefits, including in the management of bone health. This issue probably explains the north-south gradient of fractures in Europe, with a lower incidence in the Mediterranean area [2]–[4]. Indeed, Perez-Lopez et al. [5] have highlighted the benefits for bone of the Mediterranean diet (characterized by a high consumption of plant foods, low to moderate intakes of fish, poultry, dairy products and wine, a low amount of red meat, olive oil being the major source of fat, [6]) such a dietary pattern being thought to be related to the longevity of Mediterranean people. In the European Prospective Investigation into Cancer and nutrition study, increased adherence to the Mediterranean diet appears to protect against hip fracture occurrence [7]. In the same way, varied diets based on Mediterranean diet patterns have been reported to be positively associated with bone mineral density (BMD) in both pre- and post-menopausal healthy Spanish women [8]. In a holistic dietary approach, Kontogianni et al. [9] have shown that adherence to a dietary pattern close to the Mediterranean diet, i.e., high consumption of fish and olive oil and low red meat intake, was positively related to bone mass, suggesting potential bone-preserving properties of this pattern throughout adult life. It is important to know that more than 200 chemical compounds are found in olive oil, including phenols (20–500 mg/L), sterols, squalene and tocopherols [10]–[12], in addition to oleic acid (C18∶1) (55–83%), palmitic acid (C16∶0) (7.5–20%) and linoleic acid (C18∶2) (3.5–21%), as stated in the Codex Alimentarius [13]. Even though olive oil composition varies according to the cultivation area, the cultivar, the harvesting and storage systems, the ripening degree of the fruits, and the processing practices [10], [14], [15], scientists have reported that both olives [16] and olive oil [17], [18] can prevent bone loss in ovariectomized rats or mice [19]. Among olive polyphenols, oleuropein and hydroxytyrosol may have critical effects on the maintenance of bone health [17], [20]–[22]. Besides, with regards to fish consumption, it can be a major source of vitamin D (in addition to a specific content in fatty acids). Vitamin D deficiency is actually very common worldwide [23]–[25] and it is associated with a 77% increased risk of fracture in women with post-menopausal osteoporosis [26]. The pooled analysis published by Bischoff-Ferrari and coworkers concluded that high-dose vitamin D supplementation (≥800 IU daily) was somewhat favorable in the prevention of hip fracture and any non-vertebral fracture in persons 65 years of age or older [27].

As vitamin D is lipophilic, supplementation of a lipid matrix such as olive oil is feasible and might be of public interest in the management of bone health. This is why we investigated the effect of the consumption of olive oil enriched or not with vitamin D, in the ovariectomized mice as a model of post-menopausal osteoporosis. Moreover, because the bone sparing effect of the polyphenols from olive oil has already been highlighted, possible synergy between those micronutrients and vitamin D was studied and compared to the impact of a refined olive oil (depleted in polyphenols) fortified with vitamin D or not.

## Materials & Methods

### Animals

All the experimental procedures were approved by the institution’s animal welfare committee (Comité d’Ethique en Matière d’Expérimentation Animale Auvergne: CEMEAA) and were conducted in accordance with the European’s guidelines for the care and use of laboratory animals (2010- 63UE). All efforts were made to minimize animal suffering.

Six weeks old female C57BL/6J mice were obtained from Janvier (Le Genest St Isle, France) and acclimated for 2 weeks under standard laboratory conditions. They were housed individually, on a 12-h light/dark cycle, in the animal facilities of the Human Nutrition Unit at INRA Research Center (Agreement no. C6334514), with free access to both food and water. After the acclimatization period, the rodents were randomly divided into six groups (n = 12/group), although respecting similar average body weight and composition (QMR EchoMRI-900TM, Houston, USA) in each experimental group. At 8 weeks of age, 4 groups of mice were bilaterally ovariectomized (OVX) and 2 batches were sham-operated (SH) under ketamine/xylazine anesthesia, before beginning consumption of the experimental diets (Scientific Animal Food and Engineering (SAFE), Augy, France). Mice were fed a standard diet modified from the AIN-93G powdered diet. 10% of the food was given as either refined olive oil (RO) (RO-SH and RO-OVX groups) or virgin olive oil (VO) (VO-SH and VO-OVX groups). The polyphenol composition of the virgin and refined olive oil is detailed in Table 1. Moreover, 2 groups of ovariectomized mice received 5000 IU/kg diet of vitamin D3 (VD3) (RO-OVX-VD3 and VO-OVX-VD3), VD3 provided in the other diets being 1000 IU/kg diet. Food was distributed every 2 days and provided ad libitum. Food consumption, body weight and body composition were regularly monitored. After 30 days of treatment, blood was collected under anesthesia and allowed to coagulate for 30 min before separation of serum by centrifugation (4000 g, 4°C, 10 min). The serum was aliquoted and stored at −80°C until biochemical analyses were performed. After cervical dislocation, uterus, liver and spleen were weighted. The two femurs of each animal were harvested: one was rapidly frozen in nitrogen and stored at −80°C for RNA analysis, the other one was stored in 10% formaldehyde at 4°C for 5 days before being dried out for microCT analyses.

**Table 1:** Polyphenol composition of the virgin olive oil.

		Virgin olive oil		Refined olive oil
Phenolic group	Phenolic compounds	Quantity (mg/kg)	Standard deviation	Quantity (mg/kg)
Simple phenols				
	Tyrosol	14.61	0.17	n.d
	Hydroxytyrosol	14.15	0.01	n.d
Secoiridoid derivatives				
	Oleuropein-aglycone di-aldehyde	195.63	0.14	n.d
	Oleuropein-aglycone mono-aldehyde	93.55	0.29	n.d
	Ligstroside-aglycone di-aldehyde	199.22	0.26	n.d
Lignan				
	Pinoresinol	65.73	0.09	n.d
Flavonoids				
	Apigenin	1.16	0.02	n.d
	Luteolin	3.21	0.02	n.d
Total		587.26		

n.d., not detected.

### Analysis

#### Phenolic composition analysis

Extraction of phenolic compounds was carried out using one milliliter of syringic acid (0,015 mg/ml in methanol and used as internal standard) which was added at 2.0 g of olive oil before being evaporated under nitrogen. Then 6 mL of a methanol/water (80/20) mixture was added and vigorously mixed during 5 min. To finish, the extraction mixture was centrifuged at 5000 rpm during 25 min. The phenolic compounds were then quantified using an HPLC system (Agilent 1200 series) equipped with a diode array detector. Separation was performed on a column Waters Spherisorb ODS-2 (C18; 5 µm; 4.6 mm×250 mm) using a ternary gradient elution (water and 0.2% of phosphoric acid; methanol and acetonitrile). The detection was made at 280 nm and the injection volume was 20 µL.

#### Bone micro-architecture and bone mineral density analyses

Micro-architecture was investigated using X-ray radiation micro-CT (Viva CT 40, Scanco Medical, Brüttisellen, Switzerland). Scans were performed on the dried femurs at 55 keV with a 10-µm cubic resolution. The secondary spongiosa and associated cortical bone were scanned within the distal metaphasis. Trabecular bone volume (BV/TV, %), trabecular number (Tb.N,/mm), trabecular thickness (Tb.Th, µm), trabecular spacing (Tb.Sp, µm), the degree of anisotropy (DA), and the structural model index (SMI) were analyzed. Cortical thickness and porosity, bone surface and area as well as medullary area were determined as well. Moreover, BMD of the primary spongiosa was determined using an eXplore CT 120 scanner (GE Healthcare, Fairfield, CT). Acquisitions were performed with X-ray tube settings at 100 kV and 50 mA. BMD (mg/cc) was estimated as the mean converted gray-scale level within the region of interest of cortical and trabecular bone.

#### Serum Bone Turnover Markers Measurement

Serum PINP (N-terminal propeptide of type I procollagen) levels, a specific and sensitive marker of bone formation, was measured using a mouse competitive enzyme immunoassay (EIA) assay (Immunodiagnostic Systems EURL, Paris, France), according to the manufacturer’s protocol The sensitivity was 0.7 ng/mL. The intra- and inter-assay precisions were 6.4 and 9.2%, respectively.

Serum CTX-1 (collagen type 1 cross-linked C-telopeptide, a bone resorption marker) was determined using a mouse-specific enzyme-linked immunosorbent assay (ELISA) (Immunodiagnostic Systems EURL, Paris, France), according to the manufacturer’s protocol. The detection limit was 2.0 ng/mL. The intra- and inter-assay variations were 5.6 and 10.5%, respectively.

#### Taqman Low Density Array

Total RNA from powdered femurs was extracted using TRIzol reagent according to the protocol provided by the manufacturer (Invitrogen Life technology, Carlsbad, CA). After validating the RNA quality, high capacity cDNA reverse transcription kit (Applied Biosystems Life technology, Carlsbad, CA) was used to convert RNA into cDNA. Taqman Low Density Array (TLDA) (Applied Biosystems Life technology, Carlsbad, CA) was performed on reverse transcription products, using a 7900 HT Fast Real-Time PCR system (7900HT Fast Real-Time PCR system.). Gene expression was calculated relative to that of the house-keeping gene glyceraldehyde-3-phosphate dehydrogenase (GAPDH) using the comparative threshold cycle method (2^−ΔΔCT^).

Sequence references are: Gapdh;Gm12070;Gm10481-Mm03302249_g1, Trap (Acp5)-Mm00475698_m1, Alpl-Mm01187117_m1, Ocn (Bglap;Bglap-rs1;Bglap2)-Mm03413826_mH, Ccl2-Mm00441243_g1, Col1a1-Mm00801666_g1, Comp-Mm00489490_m1, Ctsk-Mm00484036_m1, Esr1-Mm00433147_m1, Il1b-Mm99999061_mH, Il6-Mm99999064_m1, Itgb3-Mm00443980_m1, Lrp5-Mm00493179_m1, Mmp2-Mm01253621_m1, Nos2-Mm00440488_m1, Pparg-Mm01184322_m1, Sfrp1-Mm03053883_s1, Sost-Mm03024247_g1, osterix (Sp7)-Mm00504574_m1, osteopontin (Spp1)-Mm00436767_m1, Tlr2-Mm00442346_m1, Tlr4-Mm00445273_m1, Rank (Tnfrsf11a)-Mm00437135_m1, Opg (Tnfrsf11b)-Mm01205928_m1, Rankl (Tnfsf11)-Mm00441908_m1.

### Statistical analysis

Data are expressed as the mean ± SEM. Homoscedasticity was checked by Levene’s test and the Grubbs test was used to identify outliers using XLStat (Addinsoft, Paris, France). Statistical analysis was performed by one-way analysis of variance (ANOVA) using XLStat. When a significant effect was detected, a post hoc Tukey test was applied to locate pairwise differences between conditions. A p value less than or equal to 0.05 was considered to be statistically significant.

## Results

The quality of the experiment was checked, notably castration efficiency was confirmed by uterine atrophy (p<0.0001) in the OVX rodents (RO-OVX: 13.0±1.4 mg; RO-OVX-VD3∶18.4±2.4 mg; VO-OVX: 12.0±1.3 mg*;* VO-OVX-VD3∶12.3±2.6 mg), compared to what was observed in the SH animals (RO-SH: 94.8±17.4 mg; VO-SH: 97.0±9.7**mg).

### Food consumption

Dietary consumption was evaluated twice during the experiment: 11 days (J11) then 23 days (J23) after the surgery. While the consumption did not statistically differ between the groups at J11 mice (3.13±0.07), surprisingly at J23, the SH mice ate significantly more than the OVX (RO-OVX: 3.29±0.12 g; RO-OVX-VD3∶3.11±0.14 g; VO-OVX: 3.22±0.11 g; VO-OVX-VD3∶3.09±0.15 g vs RO-SH: 4.05±0.22 g; VO-SH: 3.64±0.15 g; p<0.001; ANOVA).

### Body weight and body composition (Fig. 1)

**Figure 1:**
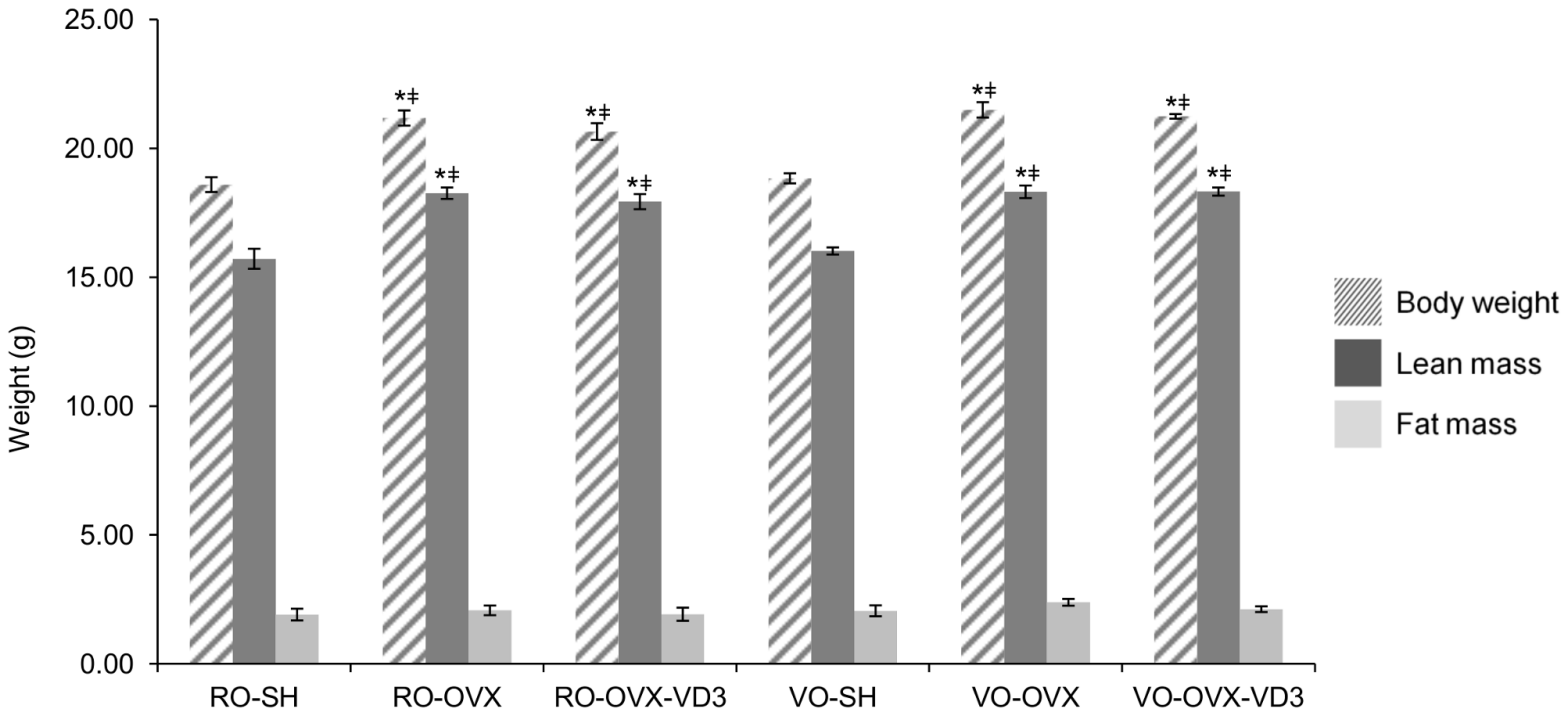
Mice body weight and composition. Body weight and composition were assessed at the end of the experiment. Values are means ± SEM. *p<0.05 vs RO-SH, ^‡^p<0.05 vs VO-SH based on ANOVA analysis with Tukey’s post hoc test. RO, refined olive oil; VO, virgin olive oil; VD3, vitamin D3; SH, sham operation; OVX, ovariectomy. Following sham operation or ovariectomy, the mice received refined or virgin olive oil for 4 weeks. Two additional groups of ovariectomized mice were given refined or virgin olive oil enriched with vitamin D3.

During the experimental period, mean body weight, as well as lean and fat mass, increased in all the experimental groups, which is a good indicator of health. At the end of the investigation, the OVX mice exhibited a higher body weight (p<0.0001) than the SH animals (RO-OVX: 21.18±0.29 g*;* RO-OVX-VD3∶20.66±0.32 g; VO-OVX: 21.50±0.30 g*;* VO-OVX-VD3∶21.24±0.09 g vs RO-SH: 18.60±0.29 g; VO-SH: 18.84±0.19 g) (Fig. 1). With regards to the body composition analysis, lean mass was significantly (p<0.0001; ANOVA) increased in the OVX groups compared to the controls whereas fat mass was similar between the groups (p>0.05; ANOVA). Therefore, body weight increases in the OVX groups are likely due to lean mass gain.

### Spleen weight

Spleen weight was evaluated as it is a primary indicator of inflammation. As expected, a splenomegaly was observed in OVX mice (RO-OVX: 71.4±3.2 mg; RO-OVX-VD3∶79.6±2.6 mg; VO-OVX: 73.2±2.7 mg; VO-OVX-VD3∶76.1±4.4 mg vs RO-SH: 57.7±2.2 mg; VO-SH: 51.4±1.7 mg; p<0.0001; ANOVA)). This parameter was not modified by any of the diet as all the OVX animals exhibited same values.

### Bone parameters (Fig. 2, table 2)

**Figure 2:**
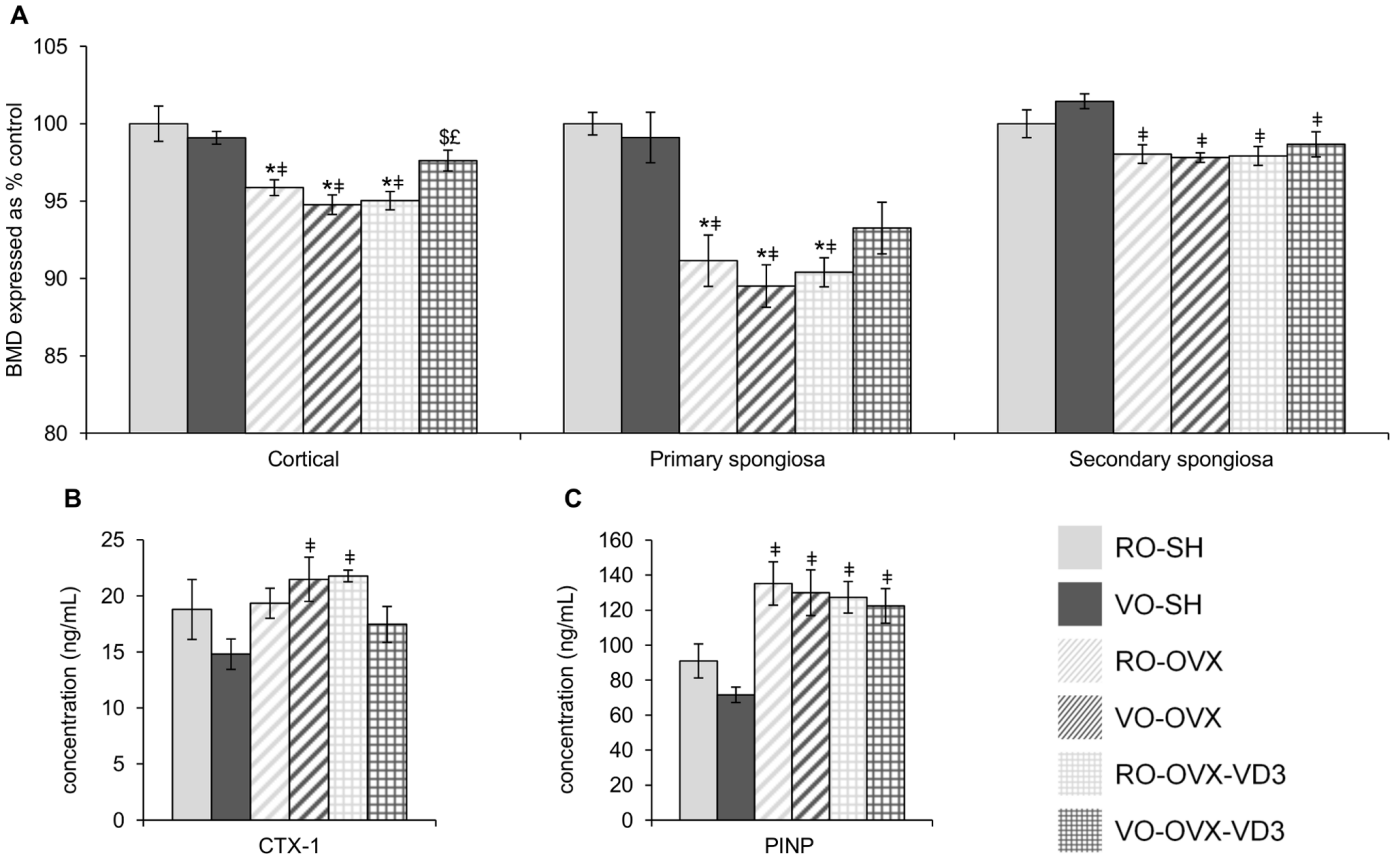
Bone parameters in mice fed with olive oil enriched or not with vitamin D3. Femoral cortical, primary and secondary spongiosa bone mineral density (A) and serum levels of CTX1 and PINP (B) in mice fed with olive oil enriched or not with vitamin D3.Following sham operation or ovariectomy, the mice received refined or virgin olive oil for 4 weeks: RO-SH, VO-SH, RO-OVX, VO-OVX. Two additional groups of ovariectomized mice received refined or virgin olive oil enriched with vitamin D3: RO-OVX-VD3 and VO-OVX-VD3. Values are means ± SEM. ANOVA with Tukey’s post hoc test were performed. *p<0.05 vs RO-SH, ^‡^p<0.05 vs VO-SH, ^$^p<0.05 vs VO-OVX, ^£^p<0.05 vs RO-OVX-VD3. RO, refined olive oil; VO, virgin olive oil; VD3, vitamin D3; SH, sham operation; OVX, ovariectomy; BMD, bone mineral density; CTX-1, collagen type 1 cross-linked C-telopeptide; PINP, N-terminal propeptide of type I procollagen.

As expected in such an animal model, BMD of the 3 compartments of interest (i.e. cortical bone, primary and secondary spongiosa) was impaired in all the OVX groups (p<0.0001; p<0.0001; p = 0.0002, respectively; ANOVA) (Fig. 2A). This goes along with altered micro-architecture, parameters such as marrow area, porosity and trabecular space being enhanced while cortical thickness, bone area and volume, as well as trabecular number being decreased compared to what was measured in the SH mice (Table 2). With regards to the diet effect, quality of olive oil did not interfere with BMD as both refined or virgin oils were devoid of any bone sparing effect (VO-OVX and RO-OVX exhibited similar values), regardless of the site considered (Fig. 2A). However, fortification of the virgin oil with vitamin D allowed to significantly mitigate cortical bone loss (VO-OVX-VD3, 1128.41±7.75 mg/cc; VO-SH, 1145.34±4.76 mg/cc vs VO-OVX, 1095.40±7.27 mg/cc; p = 0.022 and p<0.0001, respectively; ANOVA), and partially prevent bone loss at the primary spongiosa (intermediate profile between OVX and SH animals: VO-OVX, 89.51±1.37%; VO-OVX-VD3, 93.26±1.67%; VO-SH, 99.11±1.63%), while only a trend towards an improvement was observed at the secondary spongiosa (p = 0.022 between VO-SH and VO-OVX-VD3 groups; ANOVA) (Fig. 2A). On the opposite, fortification of the refined oil with vitamin D was ineffective. Finally, none of the diets was able to modify any parameter of micro-architecture.

**Table 2:** Mice femoral secondary spongiosa micro-architecture parameters.

	RO-SH	VO-SH	RO-OVX	VO-OVX	RO-OVX-VD3	VO-OVX-VD3	ANOVA
**Marrow Area (mm^2^)**	1.57±0.09	1.67±0.06	1.76±0.05	1.83±0.08	1.83±0.07	1.67±0.06	NS
**Bone Area (mm^2^)**	0.85±0.02	0.86±0.01	0.80±0.01^‡^	0.79±0.01*^‡^	0.79±0.01*^‡^	0.79±0.01*^‡^	p<0.0001
**Cortical thickness (µm)**	142±5	140±3	136±3	131±4	126±4	138±4	NS
**Porosity (%)**	7.54±0.34	7.92±0.25	8.60±0.35	8.98±0.49	8.76±0.51	7.60±0.25	NS
**Bone Surface (mm^2^)**	0.84±0.02	0.87±0.01	0.85±0.01	0.84±0.01	0.84±0.01	0.83±0.01	NS
**SMI**	2.80±0.10	2.79±0.11	2.98±0.06	3.02±0.07	2.99±0.05	2.92±0.07	NS
**BV/TV (%)**	8.32±0.84	7.87±0.85	4.88±0.38*^‡^	4.89±0.48*^‡^	5.26±0.44*^‡^	5.31±0.61*^‡^	p<0.001
**Tb.N (/mm)**	2.33±0.17	2.21±0.18	1.43±0.10*^‡^	1.45±0.12*^‡^	1.51±0.11*^‡^	1.47±0.14*^‡^	p<0.0001
**Tb.Th (µm)**	35.3±1.2	36.9±1.2	34.1±0.8	33.3±0.9	34.7±0.8	35.6±0.8	NS
**Tb.Sp (µm)**	405±33	444±42	692±46*^‡^	708±70*^‡^	667±58*^‡^	691±64*^‡^	p<0.001
**DA**	1.49±0.05	1.43±0.02	1.51±0.04	1.47±0.02	1.49±0.02	1.52±0.02	NS

Values are means ± SEM. *p<0.05 vs RO-SH, ^‡^p<0.05 vs VO-SH based on ANOVA analysis with Tukey’s post hoc test. RO, refined olive oil; VO, virgin olive oil; VD3, vitamin D3; SH, sham operation; OVX, ovariectomy, SMI, structural model index; BV/TV, trabecular bone volume; Tb.N, trabecular number; Tb.Th, trabecular thickness; Tb.Sp, trabecular spacing; DA, degree of anisotropy. Following sham operation or ovariectomy, mice received refined or virgin olive oil for 4 weeks. Two additional groups of ovariectomized mice received refined or virgin olive oil enriched with vitamin D3.

As far as biomarkers of bone metabolism are concerned, no differences were observed between the groups consuming RO (Fig. 2B and 2C). For the VO groups, the bone formation marker (PINP) was increased in the OVX groups (VO-OVX, 129.91±13.08 ng/mL; VO-OVX-VD3, 122.38±9.90 ng/mL vs VO-SH, 71.58±4.39 ng/mL; p = 0.003 and p = 0.031 respectively; ANOVA) (Fig. 2C). The bone resorption marker (CTX1) was increased in the VO-OVX group as well and has an intermediate profile in the VO-OVX-VD3 group (VO-OVX, 21.47±1.98 ng/mL; VO-OVX-VD3, 17.45±1.60 ng/mL vs VO-SH, 14.80±1.36 ng/mL; p = 0.035 and p>0.05 respectively; ANOVA) (Fig. 2B).

### Investigation of molecular changes by a TLDA analysis (Fig. 3)

A TLDA analysis allowed assessing the potential impact of the diets on a number of genes, in order to understand the involved mechanisms. Forty eight genes were investigated, therefore only the genes whose expression was different between the groups are presented. The RO-SH group was used as a reference.

#### Biological effect of ovariectomy

**Figure 3:**
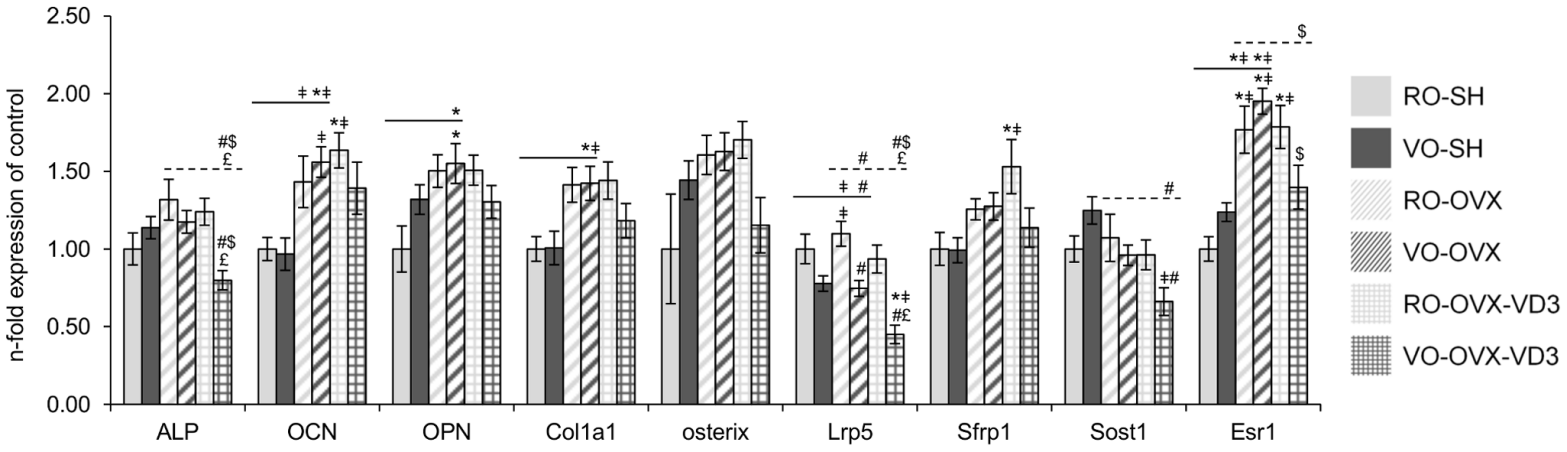
Transcript levels of osteoblast metabolism markers in mice femurs. Following sham operation or ovariectomy, the mice received refined or virgin olive oil for 4 weeks: RO-SH, VO-SH, RO-OVX, VO-OVX. Two additional groups of ovariectomized mice were given refined or virgin olive oil enriched with vitamin D3: RO-OVX-VD3 and VO-OVX-VD3. Values are means ± SEM. ANOVA with Tukey’s post hoc test were performed on the 6 groups (symbols above histograms), on the 4 groups without vitamin D3 (symbols above the solid line) and on the 4 OVX groups (symbols above the dotted line). *p<0.05 vs RO-SH, ^‡^p<0.05 vs VO-SH, ^#^p<0.05 vs RO-OVX, ^$^p<0.05 vs VO-OVX, ^£^p<0.05 vs RO-OVX-VD3. RO, refined olive oil; VO, virgin olive oil; VD3, vitamin D3; SH, sham operation; OVX, ovariectomy; ALP, alkaline phosphatase; OCN, osteocalcin; OPN, osteopontin; Col1a1, type I collagen; Lrp5, low density lipoprotein receptor-related protein 5; Sfrp1, secreted frizzled related sequence protein 1; Sost1, sclerostin; Esr1, estrogen receptor 1.

**Figure 4:**
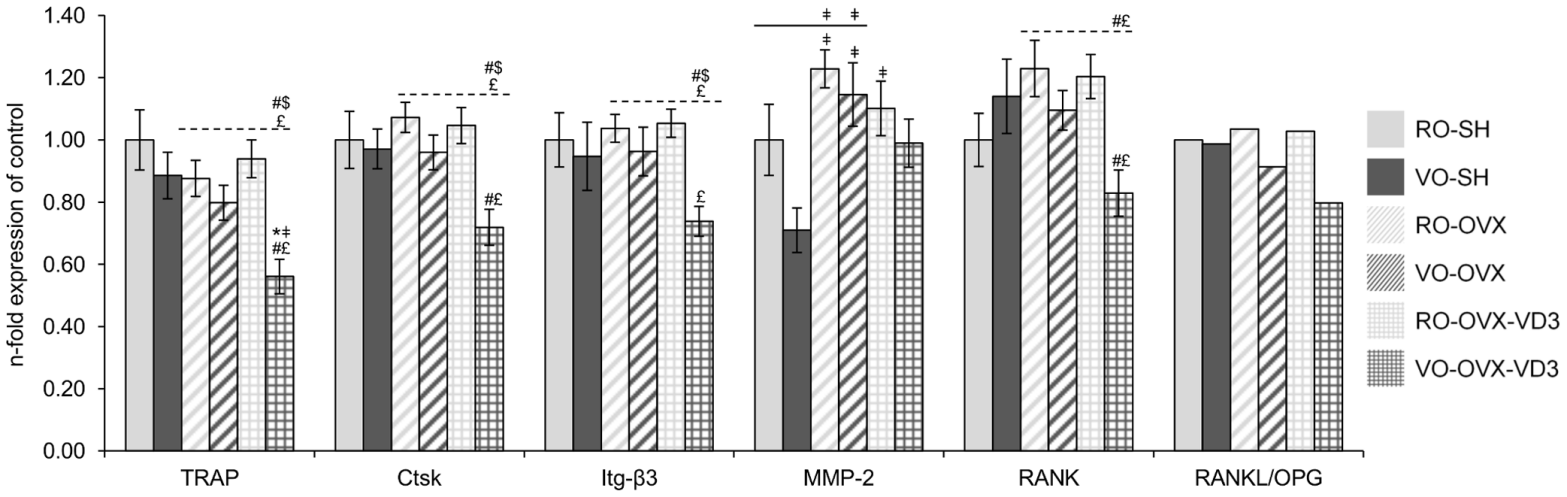
Transcript levels of osteoclast metabolism markers in mice femurs. Following sham operation or ovariectomy, the mice received refined or virgin olive oil for 4 weeks: RO-SH, VO-SH, RO-OVX, VO-OVX. Two additional groups of ovariectomized mice were given refined or virgin olive oil enriched with vitamin D3: RO-OVX-VD3 and VO-OVX-VD3. Values are means ± SEM. ANOVA with Tukey’s post hoc test were performed on the 6 groups (symbols above histograms), on the 4 groups without vitamin D3 (symbols above the solid line) and on the 4 OVX groups (symbols above the dotted line). *p<0.05 vs RO-SH, ^‡^p<0.05 vs VO-SH, ^#^p<0.05 vs RO-OVX, ^$^p<0.05 vs VO-OVX, ^£^p<0.05 vs RO-OVX-VD3. RO, refined olive oil; VO, virgin olive oil; VD3, vitamin D3; SH, sham operation; OVX, ovariectomy; TRAP, tartrate-resistant acid phosphatase; Ctsk, catepsin K; Itg-β3, β3-integrin; MMP-2, matrix metalloproteinase 2; RANK, Receptor activator of nuclear factor-kappaB (NF-kappaB); RANKL, RANK ligand; OPG, osteoprotegerin.

**Figure 5:**
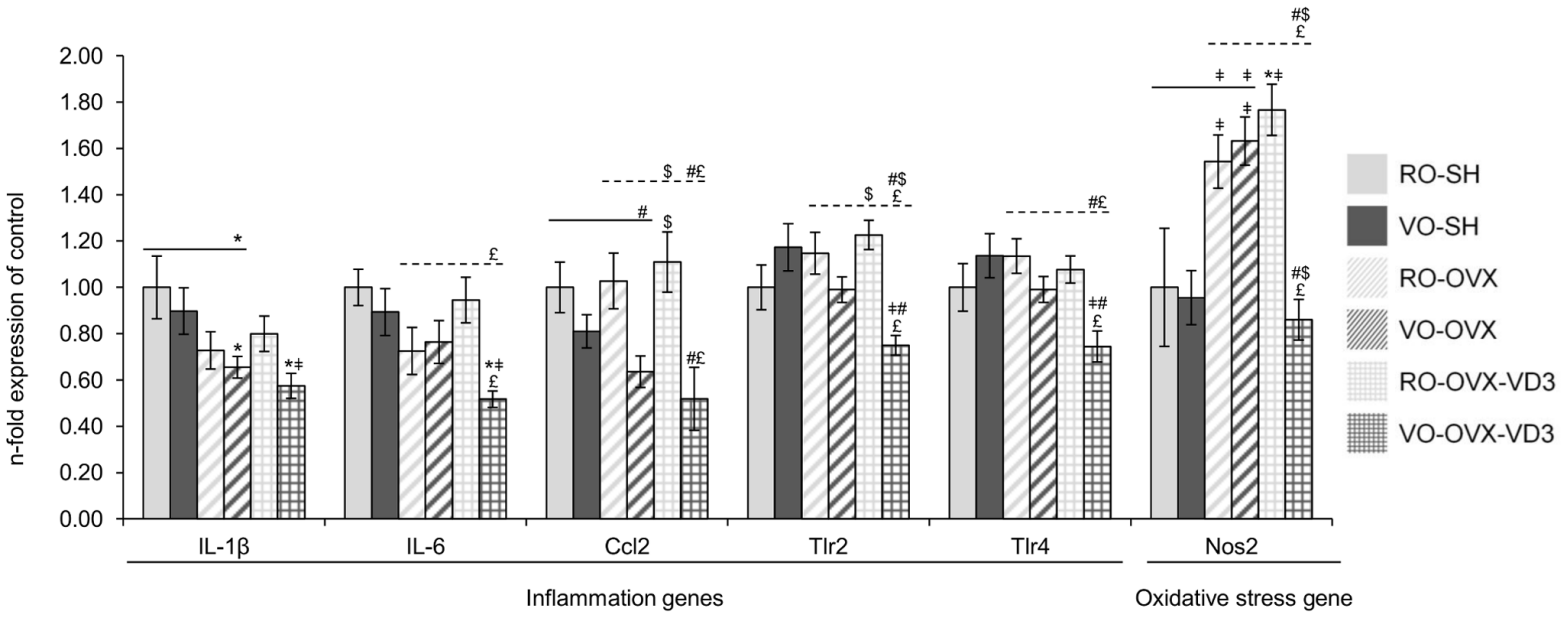
Transcript levels of oxidative stress and inflammation markers in mice femurs. Following sham operation or ovariectomy, the mice received refined or virgin olive oil for 4 weeks: RO-SH, VO-SH, RO-OVX, VO-OVX. Two additional groups of ovariectomized mice were given refined or virgin olive oil enriched with vitamin D3: RO-OVX-VD3 and VO-OVX-VD3. Values are means ± SEM. ANOVA with Tukey’s post hoc test were performed on the 6 groups (symbols above histograms), on the 4 groups without vitamin D3 (symbols above the solid line) and on the 4 OVX groups (symbols above the dotted line). *p<0.05 vs RO-SH, ^‡^p<0.05 vs VO-SH, ^#^p<0.05 vs RO-OVX, ^$^p<0.05 vs VO-OVX, ^£^p<0.05 vs RO-OVX-VD3. RO, refined olive oil; VO, virgin olive oil; VD3, vitamin D3; SH, sham operation; OVX, ovariectomy; IL-1β, interleukin-1β; IL-6, interleukin-6; CCl2, chemokine (C-C motif) ligand 2; Tlr2, toll like receptor 2; Tlr4, toll like receptor 4; Nos2, nitric oxide synthase.

Consistent with the bone biomarkers data, bone turnover genes were up-regulated in animals under estrogen deprivation. Indeed, genes representative of bone formation (osteocalcin (OCN; p = 0.0001), osteopontin (OPN; p = 0.025), type I collagen (Col1a1; p = 0.007; ANOVA without the vitamin D3 groups)) (Fig. 3) and a protease involved in the degradation of several matrix proteins (matrix metalloproteinase 2 (MMP-2)) (Fig. 4) were enhanced in the OVX rodents, compared to what was measured in the SH animals (p = 0.001;ANOVA without the vitamin D3 groups). Nevertheless, other osteoclastic markers such as catepsin K (Ctsk), tartrate-resistant acid phosphatase (TRAP) and β3-integrin (Itg-β3) stayed unchanged (Fig. 4). Besides, markers of oxidative stress were up-regulated in the OVX mice (inducible nitric oxide synthase (Nos2; p = 0.002; ANOVA without the vitamin D3 groups)) (Fig. 5). With regards to inflammation pathways, no significant difference could be observed between SH and OVX animals. It is also noticeable that expression of estrogen receptor 1 (Esr1; p<0.0001; ANOVA without the vitamin D3 groups) increased in the OVX animals compared to what was measured in the SH mice, in both diets (Fig. 3).

#### Comparison of gene expression according to the quality of olive oil (RO vs VO) in the diet

As far as bone formation is concerned, osteoblast genes were not differently expressed in the SH rodents, whether olive oil was refined or not. In OVX animals, low density lipoprotein receptor-related protein 5 (Lrp5), a receptor by which the Wnt/β-catenin signal is transduced, was less expressed in those that were given the virgin oil (p = 0.001; ANOVA without the vitamin D3 groups) (Fig. 3). Regarding osteoclast genes, no significant effect of the diet could be detected (Fig. 4). In the same way, no major effect was elicited on genes involved in both inflammation and oxidative processes (Fig. 5).

#### Synergistic effects of vitamin D and olive oil to mitigate bone loss in OVX mice

With regards to gene expression in the mice that have been given the refined oil, the only differences that were observed target OCN, secreted frizzled related sequence protein 1 (Sfrp1) and Esr1 genes (Fig. 3). Those genes were over-expressed in the RO-OVX-VD3 group compared to the RO-SH mice, but were not different from those in RO-OVX animals.

In the case of the virgin oil, fortification with vitamin D was associated to a correction of the expression of Esr1 gene (similar values in both VO-SH and VO-OVX-VD3, while the expression was higher in VO-OVX than in VO-SH) (Fig. 3). Alkaline phosphatase (ALP), Lrp5 and sclerostin (Sost1) signals were significantly reduced and there was a tendency for a reduction of osterix expression in the VO-OVX-VD3 vs the RO-OVX-VD3 group (p = 0.058; ANOVA in the OVX groups). Actually, consistently with the BMD data, the major effect targeted osteoclast genes, i.e. resorption. Indeed, the expression of TRAP, Ctsk, Itg-β3 and Receptor activator of nuclear factor-kappaB (NF-kappaB) (RANK) was down regulated by virgin olive oil and vitamin D (p = 0.0004; p = 0.001; p = 0.002; p = 0.003 respectively; ANOVA in the OVX groups), compared to what was observed in RO-OVX-VD3 mice (Fig. 4). The decreased expression of RANK was associated with a similar RANK ligand (RANKL) to osteoprotegerin (OPG) ratio. Interestingly, cartilage oligomeric matrix protein (COMP, thrombospondin 5) was significantly down regulated in VO-OVX-VD3 as compared to VO-OVX, but not statistically different from the level in VO-SH animals, elevated COMP levels in serum being found to be predictive of developing joint destruction in osteoarthritis (RO-SH: 1.00±0.10; RO-OVX: 1.32±0.15; RO-OVX-VD3∶1.42±0.10; VO-SH: 1.06±0.09; VO-OVX: 1.26±0.07; VO-OVX-VD3∶0.79±0.09; p = 0.0003; ANOVA in the 6 groups). Finally, PPAR expression, a marker for adipogenesis, was not significantly different between the experimental groups (RO-SH: 1.00±0.09; RO-OVX: 1.57±0.13; RO-OVX-VD3∶1.50±0.12; VO-SH: 1.29±0.12; VO-OVX: 1.34±0.10; VO-OVX-VD3∶1.26±0.15; p = 0.061; ANOVA in the 6 groups). Moreover, genes involved in inflammation such as IL-1β, IL-6 were significantly decreased in VO-OVX-VD3, compared to the level in VO-SH group (Fig. 5). Compared to the refined oil OVX groups, chemokine (C-C motif) ligand 2 (CCl2), Toll like receptors 2 (Tlr2) and Tlr4 were down regulated in the VO-OVX-VD3 mice (p = 0.001; p<0.0001; p = 0.001 respectively; ANOVA in the OVX groups). Finally, Nos2 expression (a marker of oxidative stress) in this test group was not different from that in VO-SH rodents, but significantly lower than in VO-OVX (p<0.0001; ANOVA in the 6 groups) (Fig. 5).

## Discussion

Broad-based preventive strategies designed to lower the risk of osteoporosis need to be implemented. This is why the concept of a healthy diet providing adequate amounts of various micronutrients deserves mention. Actually, within Europe, conspicuous differences is encountered in the severity of osteoporosis, the lowest incidence being reported in the Mediterranean area [3], where olive oil consumption is high, as well as sun shining for vitamin D. Our results, using an extensively valued model that is sensitive to various inhibitors of bone resorption used clinically [28], show that virgin olive oil exhibited synergistic bone sparing effect with vitamin D.

### Scientific value of the experimental protocol

The ovariectomized rodent is, by far, the most widely used animal model for post-menopausal osteoporosis. Actually, characteristics of skeletal physiology in the murine model share similarities with those of early post-menopausal women, in many respects. These include: increase rate of bone turnover with resorption exceeding formation, greater loss of cancellous than cortical bone; and similar skeletal response to various stimuli.

In the present study, as expected and previously shown [29], ovariectomy (confirmed by uterine atrophy) was associated with impaired femoral BMD, whatever the compartment (cortical bone, primary and secondary spongiosa). Comparative results, i.e. 4% reduction in the femoral cortical BMD with ovariectomy, were found on a similar model: 12 weeks-old C57Bl/6 ovariectomized for 4 weeks, in which mice were subjected to a standard diet [30]. This was consistent with the micro-architectural data, as indicated by raised marrow area, porosity and trabecular space, while bone area/volume, cortical thickness and trabeculae number were diminished, compared to what was observed in the SH animals. Moreover bone remodeling was accelerated in ovariectomized rats consuming virgin olive oil (increased bone biomarkers). This was corroborated by mechanistic analysis, genes involved in bone turnover being up-regulated by estrogen deprivation, as well. This increased bone remodeling, with resorption exceeding formation following ovariectomy, is well described in the literature [28], [31]. Ovarian ablation was also associated with an enhanced oxidative stress, as shown by over expression of Nos2. Estrogen deficiency is known to decrease defense against oxidative stress, which leads to bone loss [32]–[34]. Indeed, the Nos2 promoter has been shown to be under negative estrogen control [35]. This is why a stimulation of nitric oxide production by Nos2 was reported in an experimental OVX animal model and was associated with a 320% higher osteoblast apoptosis [36], [37]. In a clinical trial, F2-isoprostane levels, a marker of oxidative stress, have been negatively associated with BMD [38]. On the opposite, in OVX rats, osteopenia can be prevented by administration of a selective inhibitor of COX-2 [39]. Regarding inflammation, in the present study, ovariectomy did not elicit any significant effect on the genes involved in such a biological process, even though a splenomegaly was observed. As a matter of fact, conflicting data have been published. TGF-β1 mRNA and proteins expression levels have been reported to be significantly decreased in OVX rats compared to sham rats [40]–[42], while a significant increase in IL-1-IL-6 was observed in women with total hysterectomy and oophorectomy [43]. This was prevented in patients treated with estrogen and in those treated with estrogens and medroxyprogesteron.

### Effect of olive oil

Olive oil, whether it was refined (depleted in polyphenols) or not, was not able to prevent the increase in body weight observed in the OVX mice. This is consistent with previous data published by our team showing that neither olive oil nor oleuropein could modify this parameter in OVX rats [17], [21].

With regards to bone health, BMD was not significantly different in the OVX mice whether they were submitted to virgin or refined olive oil consumption. In other words, olive oil, when given alone, was not efficient to prevent OVX-induced osteopenia. This was consistent with the lack of significant difference in both CTX1 and OCN serum levels between those two groups. Consistently with our data, Puel et al. [17], [21] failed to see a bone sparing effect of oleuropein, olives, and olive oil. They provided evidence of efficiency of such nutrients and foods in OVX rats only when inflammation was exacerbated. The recent study conducted by Keiler et al. [44] in which a similar amount of polyphenols (10 mg/kg body weight) was evaluated corroborates our data as well. On the contrary, according to Hagiwara et al. [19] and Saleh and Saleh [18], oleuropein, hydroxytyrosol and olive oil, respectively, but not tyrosol were found to limit trabecular but not cortical bone loss induced by ovariectomy. As a matter of fact, Saleh and Saleh [18] found a positive impact of polyphenols by giving twice our and Keiler’s dose (i.e. 20 mg/kg body weight). Finally, in elderly men at high cardiovascular risk, a Mediterranean-type diet enriched with olive oil was associated with increased osteocalcin and PINP serum levels [45].

### Virgin olive oil and vitamin D3 act synergistically to prevent osteopenia on OVX mice

Ovariectomy-induced bone loss was prevented (both at the cortical and trabecular levels) by the combination of virgin olive oil and vitamin D3, but not by virgin olive oil alone, or even by refined olive oil and vitamin D. Therefore, our data support the idea that the beneficial impact of the Mediterranean diet involves, at least in part, olive oil. This food combination could be part of a strategy to restrain the development of osteoporosis and such a beneficial effect could be explained by exacerbation of the polyphenol-bone sparing properties thanks to vitamin D. Even though the impact of olive oil could be attributed to its fatty acids [46], [47], the lack of effect of the refined oil allows avoiding this hypothesis and polyphenols are thought to be at the origin of olive oil beneficial impact on bone [48]. We should not exclude the contribution of other micronutrients, such as, for example, tocopherols that are known to be decreased by refining.

**Figure 6:**
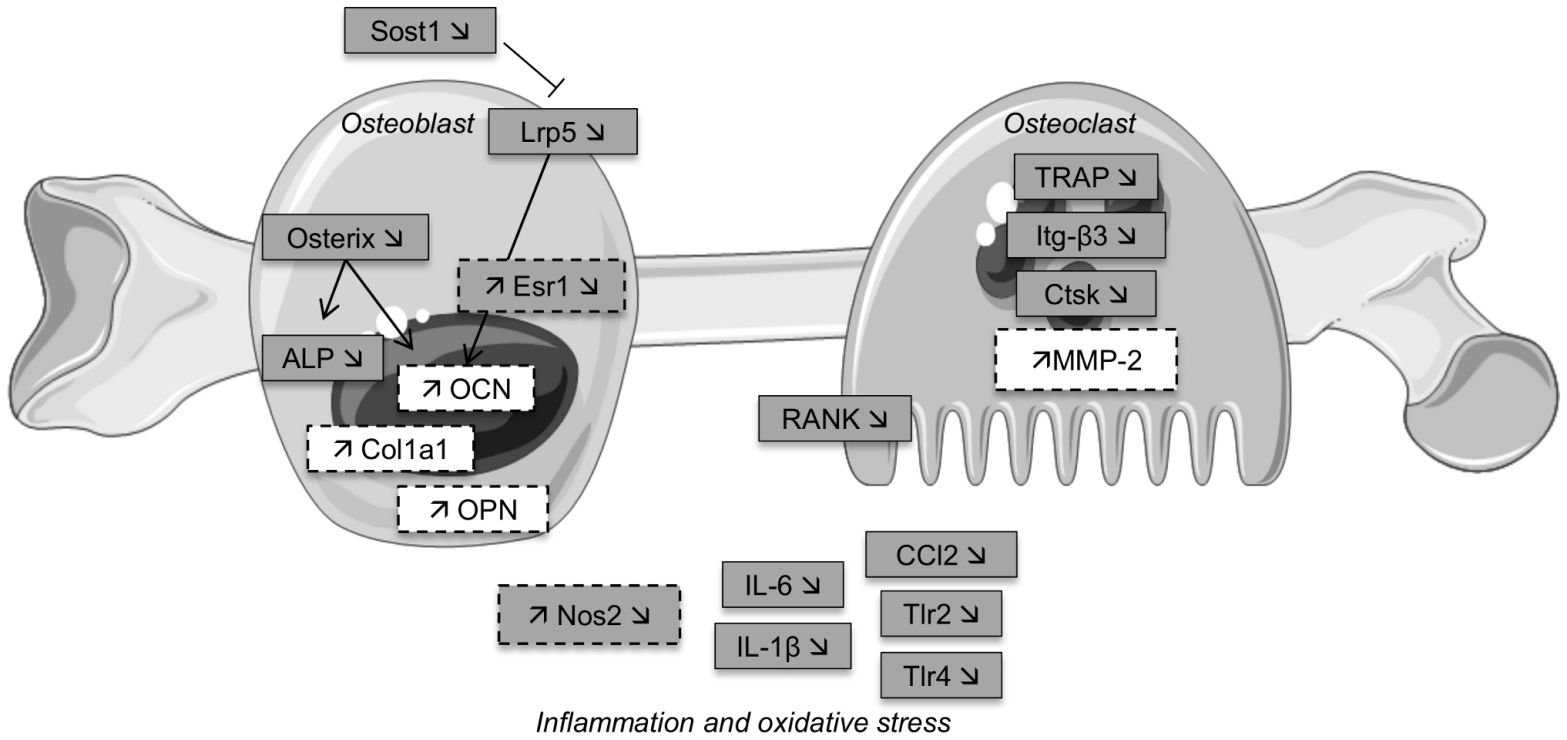
Schematic representation of the bone sparing effect of virgin olive oil enriched with vitamin D3. The effect of ovariectomy is represented by dotted boxes, the arrow (before the gene name), showing the modification of gene expression. The effect of virgin olive oil and vitamin D3 is represented by the grey boxes, the arrow (after the gene name) showing the impact on gene expression. The arrows between the genes outline regulation pathways. ALP, alkaline phosphatase; CCl2, chemokine (C-C motif) ligand 2; Col1a1, type I collagen; Ctsk, catepsin K; Esr1, estrogen receptor 1; IL-1β, interleukin-1β; IL-6, interleukin-6; Itg-β3, β3-integrin; Lrp5, low density lipoprotein receptor-related protein 5; MMP-2, matrix metalloproteinase 2; Nos2, nitric oxide synthase; OCN, osteocalcin; OPN, osteopontin; Sost1, sclerostin; Tlr2, toll like receptor 2; Tlr4, toll like receptor 4; TRAP, tartrate-resistant acid phosphatase.

In the present case, the combination of virgin olive oil and vitamin D3 indeed slowed down the ovariectomy-accelerated bone turnover. Moreover, transcriptomic analyses showed that osteoblastic and osteoclastic markers were down-regulated in VO-OVX-VD3 mice compared to the other OVX groups (Fig. 6). Therefore, bone turnover was likely to be slowed down in this group and this led to a higher primary spongiosa and cortical BMD. Oleuropein, the major olive oil polyphenol, has previously been shown to stimulate osteoblastogenesis *in vitro* by increasing the expression of Runx2, osterix, ALP and Col1a1 in mesenchymal stem cells, while adipogenesis was inhibited [22]. Besides, Hagiwara et al. [19] showed that oleuropein can increase calcium deposition by osteoblasts, and oleuropein, hydroxytyrosol and tyrosol can reduce osteoclastogenesis. On another hand, 1α,25-dihydroxyvitamin D3 has been shown to directly enhance osteoblasts mineralization [49]. Recent meta-analysis showed that vitamin D in addition to calcium can reduce the risk of hip fracture [27], [50].

The bone sparing effect observed in the animals that were given the combination of virgin olive oil and vitamin D was concomitant with decreased oxidative stress and inflammation gene expressions. Bone metabolism is impaired in the presence of an excess of reactive oxygen species, which leads to bone loss [37], [51]. The Mediterranean diet has been shown to decrease the incidence of oxidative stress which is implicated in the evolution of many diseases [52]. Moreover, polyphenols are known to have an anti-oxidant impact [53]–[55]. As a matter of fact, extra virgin olive oil and black Luckes olives were found to counteract the increased oxidative stress induced by estrogen deficiency [16], [18]. In rats which were subjected to virgin olive oil, oxidative stress parameters have been shown to be decreased in brain, skeletal muscle and cardiac muscle as well [56]–[58]. The capacity of olive oil polyphenols to decrease the expression of COX-2 and Nos2 has been described in different cell types [59]–[65].

Regarding inflammation, virgin olive oil enriched with polyphenols has been shown to exhibit protective effects in two models of inflammation [53]. Puel et al. [16], [17], [20], [21] also demonstrated that olives, olive oil, oleuropein, hydroxytyrosol and tyrosol can prevent bone loss in an inflammation model of ovariectomized rats and this was partly explained by lower plasma fibrinogen and α1-acid glycoprotein levels. It has been proposed that the anti-inflammatory impact of extra virgin olive oil could act through the NF-kappaB pathway [66]. Unfortunately, we were unable to test this gene. Our transciptomic analyses showed a protective impact of virgin olive oil associated with vitamin D3 compared to refined olive oil associated with vitamin D3 on the following markers for both oxidative stress and inflammation: Nos2, IL-6, CCl2, Tlr2 and Tlr4. In the same way, in the human study published by Fito et al. [67], IL-6 was decreased in the volunteers consuming virgin olive oil compared to refined olive oil.

In conclusion, our study shows that virgin olive oil fortified with vitamin D3 is able to counteract the bone loss induced by estrogen deprivation. Such a bone sparing effect could be explained by an improvement of both inflammation status and oxidative stress. Thus, although further data are required, virgin olive oil fortified with vitamin D might be a potential nutritional alternative for osteoporosis prevention.
